# Effects of surgical face masks on cardiopulmonary parameters during steady state exercise

**DOI:** 10.1038/s41598-020-78643-1

**Published:** 2020-12-21

**Authors:** J. Lässing, R. Falz, C. Pökel, S. Fikenzer, U. Laufs, A. Schulze, N. Hölldobler, P. Rüdrich, M. Busse

**Affiliations:** 1 Institute of Sport Medicine and Prevention, University of Leipzig, Marschnerstr. 29, 04109 Leipzig, Germany; 2 Linik Und Poliklinik Für Kardiologie, Universitätsklinikum Leipzig, Leipzig, Germany; 3 Department Sport Medicine, Institute of Applied Training Sciences Leipzig, Leipzig, Germany

**Keywords:** Physiology, Cardiology, Health care

## Abstract

Wearing face masks reduce the maximum physical performance. Sports and occupational activities are often associated with submaximal constant intensities. This prospective crossover study examined the effects of medical face masks during constant-load exercise. Fourteen healthy men (age 25.7 ± 3.5 years; height 183.8 ± 8.4 cm; weight 83.6 ± 8.4 kg) performed a lactate minimum test and a body plethysmography with and without masks. They were randomly assigned to two constant load tests at maximal lactate steady state with and without masks. The cardiopulmonary and metabolic responses were monitored using impedance cardiography and ergo-spirometry. The airway resistance was two-fold higher with the surgical mask (SM) than without the mask (SM 0.58 ± 0.16 kPa l^−1^ vs. control [Co] 0.32 ± 0.08 kPa l^−1^; p < 0.01). The constant load tests with masks compared with those without masks resulted in a significantly different ventilation (77.1 ± 9.3 l min^−1^ vs. 82.4 ± 10.7 l min^−1^; p < 0.01), oxygen uptake (33.1 ± 5 ml min^−1^ kg^−1^ vs. 34.5 ± 6 ml min^−1^ kg^−1^; p = 0.04), and heart rate (160.1 ± 11.2 bpm vs. 154.5 ± 11.4 bpm; p < 0.01). The mean cardiac output tended to be higher with a mask (28.6 ± 3.9 l min^−1^ vs. 25.9 ± 4.0 l min^−1^; p = 0.06). Similar blood pressure (177.2 ± 17.6 mmHg vs. 172.3 ± 15.8 mmHg; p = 0.33), delta lactate (4.7 ± 1.5 mmol l^−1^ vs. 4.3 ± 1.5 mmol l^−1^; p = 0.15), and rating of perceived exertion (6.9 ± 1.1 vs. 6.6 ± 1.1; p = 0.16) were observed with and without masks. Surgical face masks increase airway resistance and heart rate during steady state exercise in healthy volunteers. The perceived exertion and endurance performance were unchanged. These results may improve the assessment of wearing face masks during work and physical training.

## Introduction

During the coronavirus disease pandemic, face masks are widely recommended in medical and public areas^1,2^. Wearing face masks should reduce virus transmission^3–6^. However, the evidence of its usefulness for reducing respiratory virus infections is heterogeneous^3,7,8^. Besides its potential virus preventive effects, the use of face masks has shown increased respiratory resistance^9^. In addition, Fikenzer et al. showed a reduction in maximal physical capacity and ventilation. Occupational or physical stress may therefore be higher when using face masks and may be accompanied by an increased perception of exertion or dyspnea in patients^10^.

Respiratory protective devices or additional external breathing resistance showed similar effects on exercise capacity and ventilation^11,12^. Less marked, though comparable, results were obtained with the use of mouthguards^13–16^.

Maximum physical stress rarely occurs in occupational settings^17^. Rather, physical activity with medium or submaximal intensity is the norm. Even in leisure sports, constant-load exercise is often chosen for training. To date, no data exist on the effects of face masks on cardiopulmonary parameters during continuous exercise. Such data may allow the assessment of training and workloads associated with the use of medical masks. Specifically, possible cardiopulmonary overload due to the use of face masks could be avoided.

In summary, information on the effects of face masks during continuous exercise is lacking. Therefore, this randomized crossover study was aimed at assessing the effects of face masks on cardiopulmonary and metabolic effects at maximal lactate steady state (MLSS). Owing to the first known effects of using face masks^18^, a stronger cardiopulmonary response should be expected in the tests with masks.

## Materials and methods

### Ethical approval and study group

This study was approved by the Ethical Committee of the Medical Faculty of Leipzig University (382/20-ek) and was conducted in accordance with the latest revision of the Declaration of Helsinki. Participants were excluded from the tests if they had orthopedic, metabolic, or cardiorespiratory diseases.

Written informed consent was obtained from all participants. The study comprised 14 active and healthy men (age: 25.7 ± 3.5 years; height: 183.8 ± 8.4 cm; weight: 83.6 ± 8.4 kg; BMI, 24.7 2.6. All participants trained for at least 4 h per week. Exclusion criteria were cardiac, pulmonary, or inflammatory diseases, sports inactivity, or any other medical contraindication at the time of the examination. Participants did not perform any physical exercise 24 h before the examinations. The subjects were advised to consume a defined amount of carbohydrates (men 10 g per kg BW) within 24 h prior all tests to ensure that glycogen conditions remained stable.

### Study design

**Figure 1:**
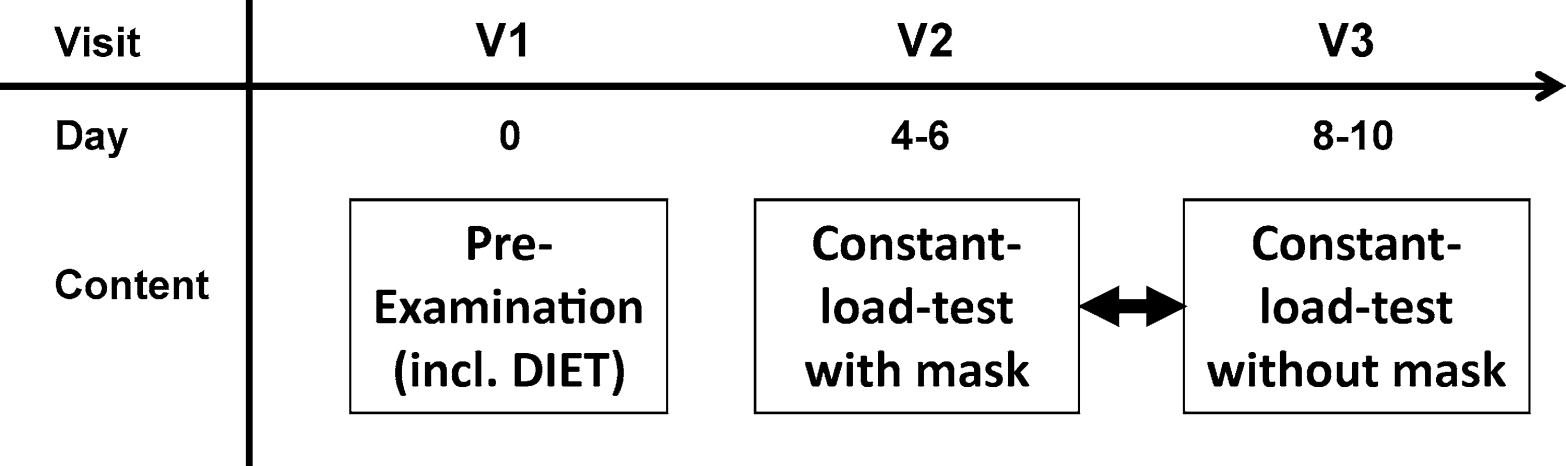
Timeline of the study; Pre-examination: informed consent, medical history, body plethysmography, *DIET* double incremental cycle ergometer test to detect the lactate minimum, Constant-load-test with and without mask: in randomized order, impedance cardiography, blood pressure, ergo-spirometry and blood lactate concentration.

A prospective, randomized, crossover design was used to examine the effects of disposable surgical face mask type II (SMs) (Suavel Protec Plus, Meditrade, Kiefersfelden, Germany) compared with no masks (control [Co]). These masks are specified by the manufacturer as Fleece 3-layer with rubber loops and an integrated nose clip. The masks were tested according to DIN EN 14683. The SM was worn under a spirometry mask (spirometric silicone masks, Cosmed, Italy) both in the body plethysmography (Fig. 1) and in the constant load tests.

The participants were tested three times during a 2-week period (pre-examination and two constant-load tests). Figure 1 presents the timeline of the study. The examination was conducted in an air-conditioning performance lab with constant temperature and humidity.

The *pre-examination* included a medical history, questionnaire (sports activity, smoking, and alcohol consumption), height and weight measurement, an electrocardiogram (Cardiax, Mesa Medizintechnik GmbH, Germany), and body plethysmography. The participants then performed a *double incremental cycle ergometer test* (DIET; the first and the second exercise period interrupted due to a 5 min recovery period) until exhaustion to assess the maximal power output (Pmax) and the MLSS due to the lactate minimum in the second load period^19,20^.

Subsequently, all participants were required to perform two constant-load tests (with and without mask) at the MLSS workload determined in the DIET in a randomized order (block randomization) at the same time of the day. The MLSS is an index of the highest oxidative metabolic rate that can be sustained during continuous exercise. Therefore, the cardiopulmonary and metabolic exposure of the participants should be comparable. All tests were performed on a semi-recumbent revolution independent cycle ergometer (Ergometrics 900, Ergoline GmbH, Bitz, Germany) at 60 to 70 revolutions per minute.

### Body plethysmography

**Figure 2:**
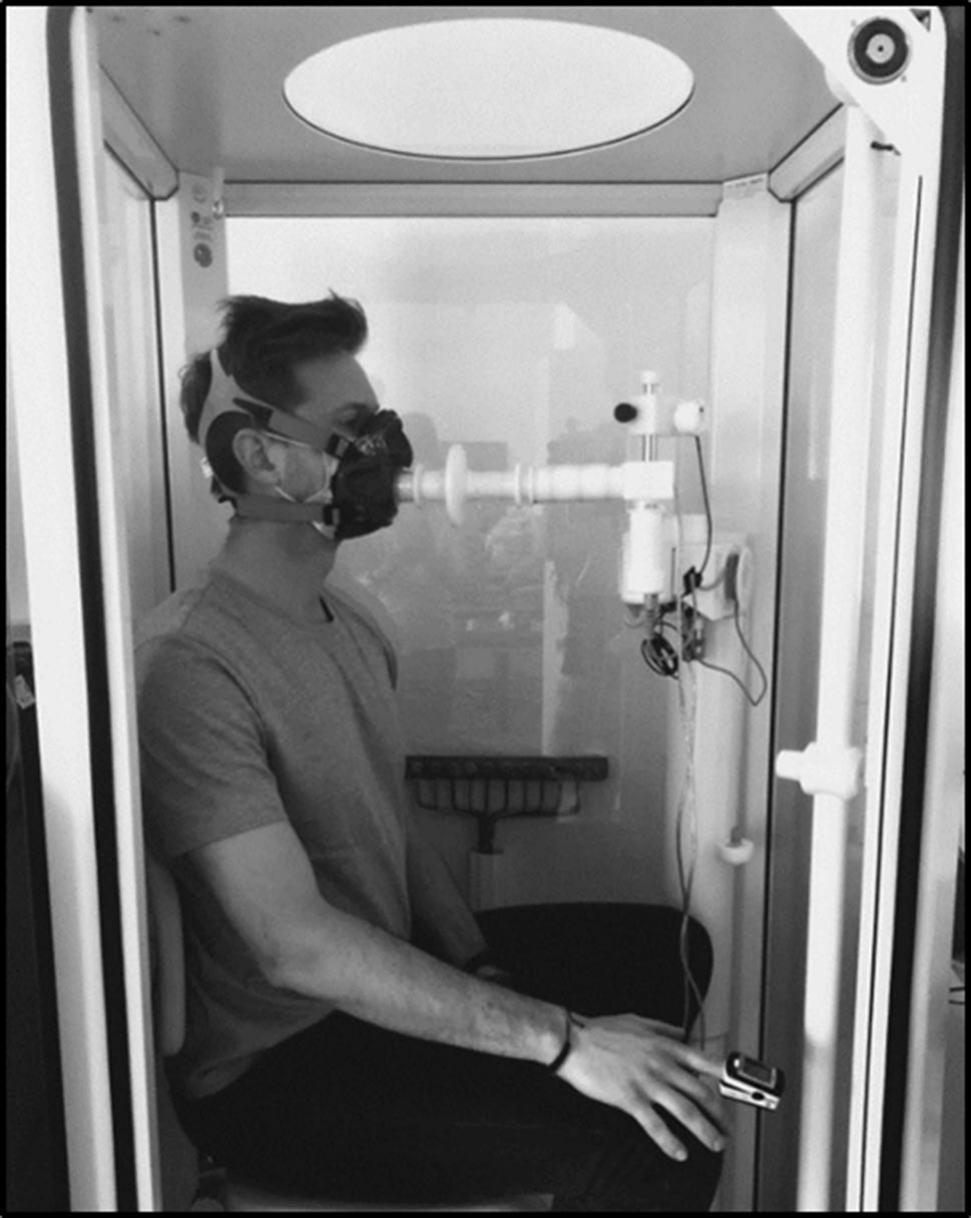
Body plethysmography measurements with spirometry masks.

Body plethysmography measurements (ZAN500 Body, nSpire Health GmbH, Germany) were performed with multi-use silicone face masks with a headgear (K4b^2^, Cosmed, Italy) (Fig. 1). The test person agreed on his written informed consent to publish the image (Fig. 2) in an online open-access publication. In addition to the standard parameters of lung function, airway resistance (R_AW_) was determined.

The examinations were performed in a randomized order with and without a surgical mask. After each test, a break of 5 min was provided to allow the breathing muscles to recover. A pulse oximeter (Pulox Fingertip Pulsoximeter, Novidion GmbH, Germany) was used to monitor possible respiratory complications.

As shown in Fig. 2, subjects were required to sit upright, keep their head in a straight line, and maintain this position for all tests. The performance of body plethysmography was standardized according to the recommendations of the German Respiratory League and the German Society for Pneumology and Respiratory Medicine^21^.

### Double incremental exertion test (DIET)/lactate minimum test

The DIET to determine the maximum load and the lactate minimum was performed without a SM. The test participants started the step test at 50 W and increased by 15 W min^−1^ until the maximum load was reached. The maximum wattage value in the first part of the DIET was assumed to be the achieved wattage, which was cycled for one min with a minimum frequency of 60 revolutions per minute. This was followed by a 5-min active recovery phase during which 25% of the maximum power was used, followed by the second part of the step test (increment: 15 W min^−1^) up to the maximum possible load. Spirometry (K4b^2^, Cosmed, Italy), thoracic impedance (PhysioFlow, Manatec Biomedical Inc., France), and a vector electrocardiogram (Cardiac PC-EKG, MESA Medizintechnik GmbH, Germany) were synchronized and run simultaneously during the complete time. Blood lactate samples (20 µL; analyzed by Super GL, Dr. Müller Gerätebau GmbH, Germany), blood pressure (Riva – Rocci) (BP), and the rating of perceived exertion (RPE) were recorded every 3 min in the initial segment of the DIET. During the second part of the step test, lactate was measured every minute. At the end of the test, lactate was measured in the first, third, and fifth minutes. The lactate values of the two-step test were represented as third-order polynomial function, and the physiological lactate minimum test was determined as described by Tegtbur et al.^20,22^. The individually determined MLSS and the wattage thus determined were used as workload for the two constant load tests.

### Constant-load tests (30 min)

Prior to the test, 5 min were recorded to determine the resting values. The constant-load tests were performed after a 5-min warm-up phase at 50% of the maximal workload in the DIET. Subsequently, the test subjects were required to cycle the determined workload in the DIET at MLSS for 30 min at a minimum frequency of 60 rpm. The two tests were performed randomly with and without a face mask. The surgical masks were worn under the spirometry mask and were applied immediately prior to exercise. Spirometry and thoracic impedance measurements were synchronized and recorded simultaneously during the entire exercise period.

### Measurements during the constant-load tests/calculations

Cardiac output (CO), stroke volume (SV), and heart rate (HR), measured using impedance cardiography; (Physioflow, Manatec Biomedical, Macheren, France), oxygen consumption (${\text{VO}}_{2}$), and respiratory parameters (minute ventilation [V_E_], tidal volume [V_T_], respiratory rate [RR]) were monitored continuously at rest, during stress, and during recovery (K4b^2^, Cosmed, Italy). Capillary blood samples (20 µl) to measure the blood lactate concentration, blood pressure (Riva-Rocci method), oxygen saturation (Pulox Fingertip Pulsoxymeter, Novodion GmbH, Germany), and RPE (from 1 to 10, if 10 was total exhaustion) were observed at rest and every 5 min during each constant load test and at 1, 3, and 5 min of recovery. Blood samples were drawn from the earlobe and analyzed immediately via the enzymatic-amperometric method (Super GL, Dr. Mueller Geraetebau GmbH, Freital, Germany). The arteriovenous oxygen difference (avDO_2_) was calculated using Fick's principle with avDO_2_ = oxygen uptake (${\text{VO}}_{2}$)/CO. Cardiac work (CW) was measured in J and calculated according to the formula CW = SV × systolic blood pressure (SBP). The MLSS in the constant-load tests was maintained if the lactate concentration did not increase by more than 1 mmol l^−1^ in the last 20 min of the minimum test^22^. The displayed lactate concentration change over time (LAC∆) is the average during 30 min of exercise minus the rest lactate. The calculation of alveolar ventilation (${\dot{\text{V}}}_{A}$) was performed according to the spirometrically recorded parameters, which were used in the following calculations (Bohr formula):

$${\dot{\text{V}}}_{A} = \left( {{\text{V}}_{\text{T}} - {\text{VD}}} \right)*{\text{RR}}$$

where ${\dot{\text{V}}}_{A}$ = alveolar ventilation; V_T_ = tidal volume; VD = dead space volume; RR = respiratory rate.

$${\text{VD}} = {\text{V}}_{\text{T}} *{\text{FetCO}}_{2} \, - \left( {{\text{FeCO}}_{2} /{\text{FetCO}}_{2} } \right)$$

where FeCO_2_ = fractional carbon dioxide concentration; FetCO_2_ = end-tidal fractional carbon dioxide concentration.

### Statistical analyses

All values are presented as means with standard deviation. GraphPad Prism 8 (GraphPad Software Inc., California, US) was used for the statistical evaluation and preparation of graphs. The values of the maximum power output (DIET) was based on the last load step of the first part of DIET and are only shown descriptively (no statistical analysis). For statistical analysis, the continuously collected data of spirometry and thoracic impedance and the punctual measurements of blood pressure, blood lactate concentration, rating of perceived exertion, and oxygen saturation were averaged for the exercise period (30 min constant load). The data were assessed for outliers using the "Rout Method". The False Discovery Rate (FDR) was specify with Q = 0.5%. The mean values of all parameters were assessed for a normal distribution using the Kolmogorov–Smirnov test. If a normal distribution was evident, statistical comparisons were made using a paired Student’s t-test (parameters of body plethysmography and mean values of constant load test). The significance level was set at α = 0.05. Non normal distribution parameters were analyzed with the Wilcoxon Matched-Pairs Signed Ranks test (parameter: RPE).

## Results

### Results of body plethysmography

Body plethysmography analyses showed significant differences among the lung function parameters (Table 1).

**Table 1:** Results of body plethysmography.

	Co	SM	p-value	n^2^_p_
FEV_1_ (l)	4.66 ± 0.61	4.18 ± 0.69	< 0.01*	0.84
PEF (l s^−^1)	9.50 ± 1.19	8.07 ± 1.25	< 0.01*	0.71
R_AW_ (kPa l^−^1)	0.32 ± 0.08	0.58 ± 0.16	< 0.01*	0.79
VC (l)	6.00 ± 0.72	5.65 ± 0.65	< 0.01*	0.82

The values are given as means and standard deviations.

*Co* control; *SM* surgical mask; *FEV*_*1*_ forced expiratory volume in 1 s; *PEF* peak expiratory flow; *RAW* airway resistance; *VC* vital capacity; *n*^*2*^_*p*_ partial eta-squared.

*Significant differences from the control.

The respiratory work were calculated from the peak flow and airway resistance (R_AW_) and showed significant differences (Co 3.0 ± 0.7 kPa vs. SM 4.6 ± 1.2 kPa*, p* < 0.01, n^2^_p_ = 0.71).

### Incremental exertion test/lactate minimum test

The maximum values of DIET are shown in Table 2. The average duration of DIET was 17:42 ± 2:42 min, and the participants reached an average Pmax of 300.7 ± 40.5 W, which corresponds to a relative power of 3.70 ± 0.63 W kg^−1^. The lactate minimum in this test was 202.69 ± 25.95 W with an LAC of 6.48 ± 1.35 mmol l^−1^.

**Table 2:** Mean values with and without SMs during the 30 min continuous tests (n = 13; excluding the warm-up and recovery phase).

	Co	SM	p-value	n^2^_p_	DIET
**Pulmonary parameters**					
${\text{VO}}_{2}$(ml min^−1^ kg^−1^)	34.49 ± 5.79	33.05 ± 4.96	0.04*	0.31	44.79 ± 9.12
${\dot{\text{V}}\text{CO}}_{2}$ (ml min^−1^)	2685 ± 278	2575 ± 310	0.04*	0.30	3958 ± 560
V_E_ (l min^−1^)	82.42 ± 10.66	77.05 ± 9.26	< 0.01*	0.61	141.83 ± 22.73
${\text{V}}_{E}/{\dot{V}}{\text{O}}_{2}$ (l min^−1^/l min^−1^)	29.34 ± 4.41	28.50 ± 3.42	0.19	0.14	38.75 ± 7.12
${\text{V}}_{E}/\dot{V}{\text{CO}}_{2}$ (l min^−1^/l min^−1^)	30.80 ± 3.81	30.01 ± 3.33	0.26	0.10	35.90 ± 3.87
${\dot{\text{V}}}_{{\text{A}}}$ (l min^−1^)	60.88 ± 7.48	57.38 ± 6.55	0.01*	0.42	102,65 ± 17,71
T_i_ (s)	0.89 ± 0.14	1.01 ± 0.13	< 0.01*	0.73	0.63 ± 0.11
T_e_ (s)	0.98 ± 0.21	0.95 ± 0.17	0.14	0.17	0.66 ± 0.17
V_T_ (l)	2.49 ± 0.35	2.45 ± 0.36	0.48	0.04	2.98 ± 0.59
RR (bpm)	34.03 ± 7.29	32.09 ± 5.40	0.02*	0.37	49.08 ± 11.23
**Hemodynamic parameters**					
SBP (mmHg)	172.3 ± 15.8	177.2 ± 17.6	0.33	0.08	210.4 ± 26.9
DBP (mmHg)	74.6 ± 6.4	72.3 ± 9.1	0.20	0.13	77.5 ± 13.7
CO (l min^−1^)	25.93 ± 4.04	28.59 ± 3.94	0.06	0.27	32.06 ± 4.62
SV (ml)	168.4 ± 30.87	178.8 ± 25.67	0.22	0.12	176.02 ± 26.91
HR (bpm)	154.5 ± 11.4	160.1 ± 11.2	< 0.01*	0.59	183.0 ± 11.4
CW (J)	29,049 ± 6165	31,866 ± 6770	0.14	0.17	37,040 ± 6964
avDO_2_ (%)	11.11 ± 1.84	9.59 ± 1.44	0.02*	0.38	11.74 ± 2.13
LAC∆ (mmol l^−1^)	4.27 ± 1.46	4.71 ± 1.42	0.26	0.11	9.01 ± 1.79
RER	0.95 ± 0.05	0.95 ± 0.05	0.97	–	1.08 ± 0.11
RPE (1–10)	6.6 ± 1.1	6.9 ± 1.1	0.16	0.16	10
SO_2_ (%)	95.22 ± 0.71	95.32 ± 0.83	0.73	0.01	–
Mean power output (Watt)	202.7 ± 26.0	202.7 ± 26.0	1.0	–	300.71 ± 40.52
Exercise duration (min)	30 ± 0	30 ± 0	1.0		–

Values are given as the means and standard deviations. The values are presented as means and standard deviations.

*DIET* double incremental exercise test; *Co* control; *SM* surgical mask; *IET* incremental exertion test; ${\text{VO}}_{2}$ oxygen uptake; $\dot{V}CO_{2}$ carbon dioxide production; *V*_*E*_ minute ventilation; $V_{E}/{V}O_{2}$ refers to the number of liters of ventilation per liter of oxygen consumed; $V_{E}/\dot{V}CO_{2}$ refers to the number of liters of ventilation per liter of carbon dioxide ${\dot{\text{V}}}_{A}$ alveolar ventilation; *RR* respiratory rate; *V*_*T*_ tidal volume; *T*_*i*_ inspiratory time; *T*_*e*_ expiratory time; *SBP* systolic blood pressure (5-min interval); *DBP* diastolic blood pressure (5-min interval); *SV* stroke volume; *CO* cardiac output; *HR* heart rate; *avDO*_*2*_ arteriovenous oxygen difference; *RER* respiratory exchange ratio, *LAC* blood lactate concentration (5-min interval); *SO*_*2*_*%* oxygen saturation *RPE* rating of perceived exertion (5-min interval); n^2^_p_.

*Significant differences from control.

### Constant load tests

**Figure 3:**
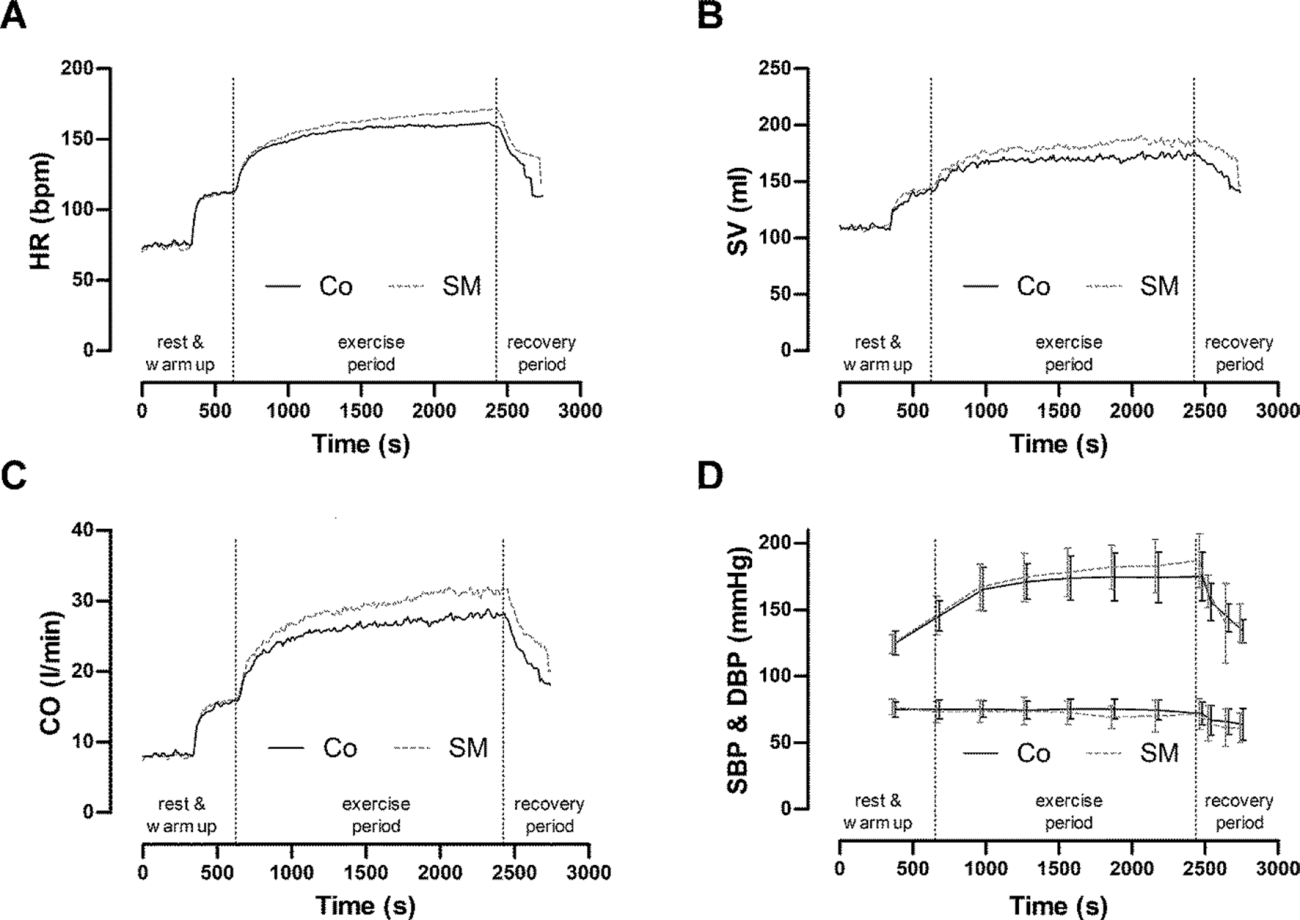
Graphs showing the mean cardiac responses (n = 13) during the continuous load test with (SM) and without (Co) a surgical mask. (**A**) HR: heart rate; (**B**) SV: stroke volume; (**C**) CO: cardiac output; (**D**) SBP & DBP: systolic & diastolic blood pressure. Rest values were determined immediately prior to exercise.

There were visible changes resting values for V_E_, BP, CO, and inspiration time (T_i_) (V_E_: Co 13.82 ± 5.79 l min^−1^ vs. SM 10.14 ± 2.78 l min^−1^; RR: Co 19.44 ± 4.33 bpm vs. SM 16.58 ± 4.90 bpm; T_i_: Co 1.34 ± 0.21 s vs. SM 1.63 ± 0.31 s). The resting lactate values were lower with SMs than with Co (Co 0.92 ± 0.26 mmol l^−1^ vs. SM 0.79 ± 0.25 mmol l^−1^). There were no visible differences in the hemodynamics. Figure 3 shows the time course of HR, CO, SV, and BP in the two constant-load tests. Table 2 shows the results of the constant-load tests. Thirteen participants completed 30 min of exercise in both tests. One test participant could not perform the test with a SM because of subjectively perceived breathing distress.

Table 2 shows the average values of the constant load tests (30 min) and the peak values of the DIET.

## Discussion

**Figure 4:**
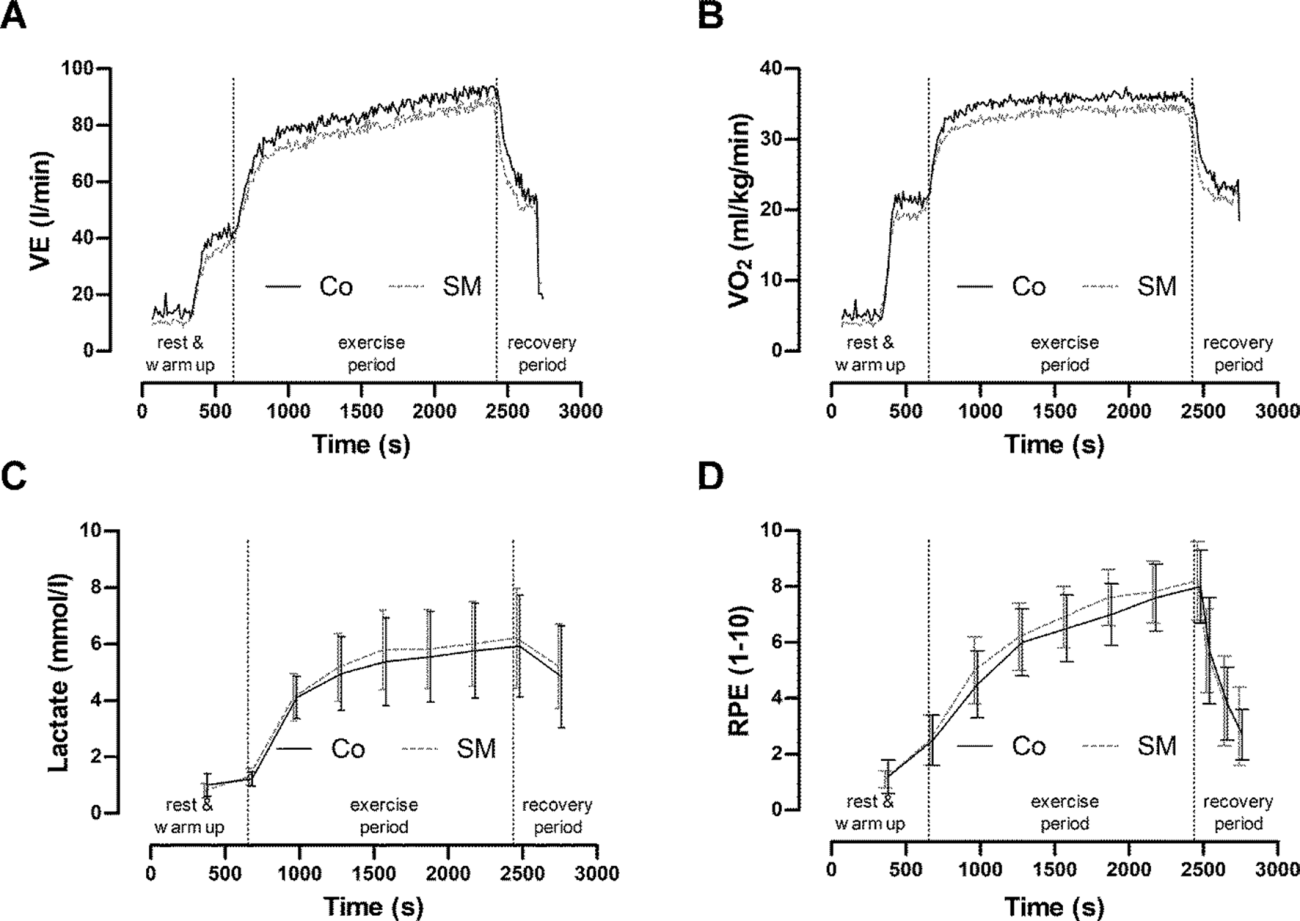
The graphs show the mean cardiac responses (n = 13) during the continuous load test with and without a surgical mask. (**A**) VE: minute ventilation; (**B**) VO_2_: oxygen uptake; (**C**) LAC: blood lactate concentration; (**D**) RPE: rating of perceived exertion (**D**). The rest values were determined immediately prior to exercise.

The main finding of this randomized crossover study was that the use of SMs during constant exercise was associated with significant changes in the values of the pulmonary and cardiac parameters as compared without the use of face masks (Figs. 3 and 4). Body plethysmography revealed a two-fold higher R_AW_ when masks were used. However, the ${\text{VO}}_{2}$ and avDO_2_ was reduced when SMs were used. Despite these cardiopulmonary changes, the constant load tests at maximal lactate steady state were completed with exception of one test subject when using masks.

### Pulmonary function

Body plethysmography revealed an increased R_AW_ and reduced forced expiratory volume in 1 s, V_C_, and peak expiratory flow with SMs. Respiratory protective devices and respiratory filters have similar effects^9,11^. Exercise studies showed that an increased R_AW_ can also significantly reduce V_E_ under stress and can decrease work performance^11,12,16,18^. Similar results were also shown in studies with mouthguards where a slightly increased R_AW_ and reduced respiratory parameters were observed at rest and during exercise^16^. A reduced RR with corresponding changes in Ti was observed. The expiratory time and V_T_ were not affected by the use of masks. The extended T_i_ is probably a compensatory mechanism that stabilizes V_T_ under these conditions^13,14^. Francis and Brasher assumed that a mechanism similar to the “pursed lip” type of breathing in pulmonary obstructive patients extends the respiratory cycle time and thus promotes gas exchange. As a result, the alveolar ${\dot{\text{V}}}_{{\text{A}}}$ is significantly reduced by using a mask. Therefore, the displayed reduction in oxygen uptake is expected.

However, the increased R_AW_ values lead to a changed exercise breathing pattern in healthy volunteers with a slowed restriction of ${\text{VO}}_{2}$^11,18^. We can assume that patients with pulmonary obstructive disease are exposed to far greater restrictions than when wearing a SM^10^. These results show a clear effect of wearing SMs on pulmonary parameters at rest and during exercise (Fig. 4).

### Cardiac function

In the present study, the use of SMs resulted in a significantly increased HR, a physiologically substantial nevertheless not significant increased SV, and tendency toward an increased CO. The increased HR during constant-load exercise with a mask might be the result of increased work of breathing or muscle affarences^19^. By contrast, other studies with oxygen-enriched air in patients with chronic obstructive pulmonary disease showed a decrease in heart rate^23^. Furthermore, increased respiratory muscle work due to reflex mechanisms could also be responsible for the increase in CO^9,24^.

Ryan et al.^25^ showed that under resting conditions, increased R_AW_ can lead to a significant increase in SV. The observed prolonged T_i_ and increased R_AW_ suggest a change in pulmonary regulation when using a SM. Prolonged or higher negative pleural pressure is assumed to improve the transmural pressure difference of the extrathoracic and intrathoracic vessels^25,26^. This may increase venous blood return and improve SV^16,18,25–27^. Use of a mouthguard increased the R_AW_, prolonged the T_i_, and increased the SV during physical stress^16^. Similar results were reported in a study by Fikenzer et al.^18^, which showed a trend toward an increased SV when using a SM, with a reduced V_E_ and significantly extended T_i_. In contrast to the present results, Fikenzer et al. showed significantly lower maximum heart rate values when a SM was used during incremental exercise. However, constant load exercise was performed in the present study, and the results may therefore not be comparable.

### Perceived exertion and metabolic response

The subjective perceived stress (Borg scale) showed no significant difference between the performances during the constant load tests. During incremental exercise, the difference in perceived stress and performance with and without masks was determined^18^. A limitation of the perceived and completed performance due to the wearing of SMs was not observed in our study. The LAC∆ tended to be higher (10.6% not statistically significant) and avDO_2_ was reduced when SMs were used.

The reduced avDO_2_ in physical stress is consistent with the findings of other studies^18^ with face masks. Presumably, ${\text{VO}}_{2}$ was lower because of the decreased ${\dot{\text{V}}}_{A}$. The reduced tissue oxygenation due to ventilatory obstruction was speculated to be responsible for the higher lactate values and cardiac drive from the working muscles^19^. The present results refer to young healthy men and can therefore be considered as a reference for this cohort.

### Limitations of the study

The study group was small and consisted of healthy men. Therefore, the data cannot be transferred to other populations. Thus, an assessment of the effect of face masks in older people and in patients with lung and heart diseases is limited. This study is the first crossover study to date that compared the acute cardiopulmonary effects during constant load (MLSS) with and without medical face masks. The cardiac parameters measured using impedance cardiography can be overestimated with absolute values^28^. However, thoracic impedance cardiography is well established for the quantification of individual changes in SV and CO^29^. The fact that the SM was worn under the spirometry mask because of a definitive seal between the SM and the face must also be taken into account. This changes the natural position of the SM and might have influenced the results.

## Conclusion

In the healthy young men (age, 25.7 ± 3.5 years) in this study, the use of surgical face masks was associated with a significant increase in airway resistance, reduced oxygen uptake, and increased heart rate during continuous exercise. Despite these changes, the endurance performance and perceived stress remained unchanged as compared with the performance without a SM. These data are useful for the assessment of the effects of SMs in occupational and sports settings. Further studies in the elderly and in persons with pulmonary or cardiac diseases are necessary.

## Abbreviations

avDO_2_: Arteriovenous oxygen difference
BP: Blood pressure
CO: Cardiac output
Co: Control
CW: Cardiac work
DIET: Double incremental cycle ergometer test
FetCO_2_: End-tidal fractional carbon dioxide concentration
HR: Heart rate
LAC: Blood lactate concentration
LAC∆: Lactate concentration change over time
MLSS: Maximal lactate steady state
SM: Surgical mask
PMAX: Maximal power output
R_AW_: Airway resistance
RR: Respiratory rate
SBP: Systolic blood pressure
SV: Stroke volume
SO_2_: Oxygen saturation
Ti: Inspiratory time
VC: Vital capacity
$${\dot{\text{V}}}_{A}$$: Alveolar ventilation
VD: Dead space volume
VE: Minute ventilation
${\text{V}}_{E}/{\text{VO}}_{2}$: Refers to the number of liters of ventilation per liter of oxygen consumed
${\text{V}}_{E}/{\dot{\text{V}}\text{CO}}_{2}$: Refers to the number of liters of ventilation per liter of carbon dioxide
$${\text{VO}}_{2}$$: Oxygen uptake
VT: Tidal volume
W: Watts
